# A Biophysical Model for Simultaneous Cochlear and Vestibular Nerve Stimulation: Insights into Neural Activation and Crosstalk in Inner Ear Implants

**DOI:** 10.1007/s10162-026-01065-9

**Published:** 2026-07-24

**Authors:** Björn Vey, Michael Handler, Rudolf Glueckert, Rami Saba, Carolyn Garnham, Daniel Baumgarten

**Affiliations:** 1 Biomedical Engineering Group, Department of Mechatronics, University of Innsbruck, Innsbruck, Austria; 2 Institute of Electrical and Biomedical Engineering, UMIT TIROL - Private University for Health Sciences and Health Technology, Hall in Tirol, Austria; 3 Department of Otolaryngology, Medical University of Innsbruck, Innsbruck, Austria; 4 Tirol Kliniken, University Clinics Innsbruck, ENT Clinic, Innsbruck, Austria; 5 MED-EL GmbH, Innsbruck, Austria

**Keywords:** Cochlear implant, Vestibular implant, Cochlear-vestibular implant (CVI), Neural activation modeling, Finite element method (FEM), Electrical stimulation, Cross-talk, Inner ear neuroprosthetics, Computational neuroscience

## Abstract

**Purpose:**

Simultaneous hearing and balance restoration through combined cochlear-vestibular implants (CVIs) offers a promising treatment for patients with dual sensory deficits. However, the effects of electrical stimulation on neighboring neural structures in the inner ear remain poorly understood.

**Methods:**

In this study, we present a detailed computational model of the human inner ear that simulates electrical stimulation of cochlear and vestibular nerves under clinically relevant conditions. The model integrates high-resolution micro-CT-based geometry, anisotropic tissue conductivities, and myelinated fiber models to predict neural activation patterns across a wide range of clinically relevant stimulation parameters.

**Results:**

Simulation results suggest that vestibular stimulation at clinically relevant amplitudes can influence cochlear nerve activation thresholds, particularly in basal cochlear regions. Conversely, cochlear stimulation had a comparatively weaker effect on vestibular activation. Interleaved stimulation with short interstimulus intervals (smaller 100 μs) resulted in increased excitability in the non-targeted nerve population, suggesting the potential for undesired cross-talk effects. Pulse waveform characteristics, including phase duration and symmetry, further modulated the degree of crosstalk observed.

**Conclusion:**

This model allows for comprehensive evaluation of scenarios that cannot be tested in humans due to ethical, practical, or technical limitations. As a result, it provides a valuable tool for exploring new combined simulation scenarios and may aid the development of newly designed implants.

## Introduction

Hearing loss significantly affects the quality of life, impacting physical, cognitive, and mental health [1]. A recent study conducted by Ihler et al. [2] suggests a 14.2% prevalence of moderate to severe hearing loss in adults. The treatment with cochlear implants (CIs) offers a well-established solution for sensorineural hearing loss by stimulating surviving cochlear neurons to replicate external sound patterns. An electrode array within the cochlea enables frequency-specific stimulation, re-enabling the patient’s hearing ability. The adjacent vestibular system plays a crucial role in balance and spatial orientation, with dysfunctions in this system severely reducing the quality of life for approximately 6.5–7.4% of adults [3]. Another recent study conducted by Hackenberg et al. [4] has shown that vertigo alone has a prevalence of 21.6% in adults, with an 8.1% prevalence considering patients with high to very high distress caused by the disease. Vestibular implants (VIs) have shown promise in both animal and human studies, partially restoring function by stimulating ampullary nerves [3, 5, 6].

**Fig. 1:**
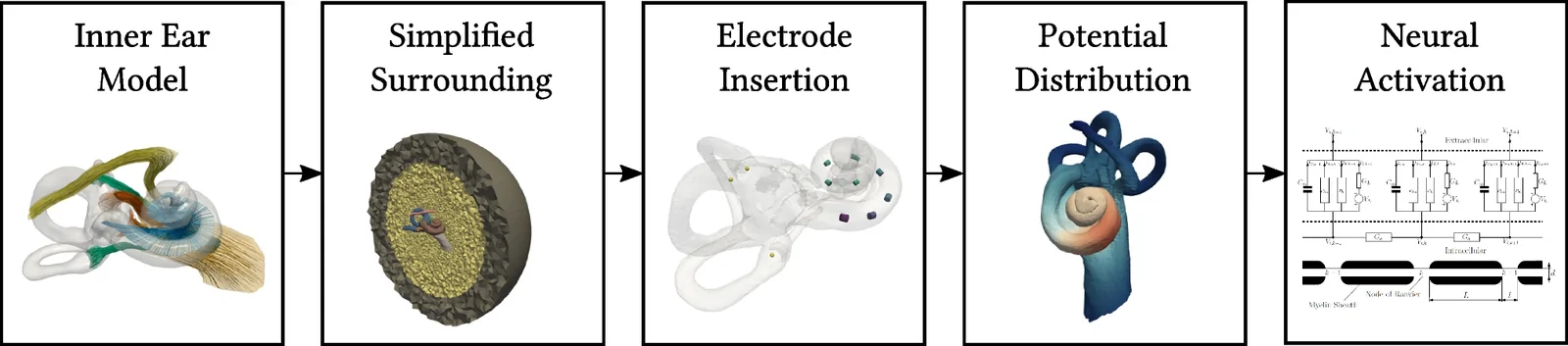
Workflow used for nerve activation threshold activation simulations carried out in this work. Some of the steps were adapted after [18, 19] as described further in the text

The complexity of combined inner ear disorders, such as those seen in Usher syndrome or hearing loss following meningitis, highlights the need for integrated cochlear-vestibular implants (CVIs) [7], as targeted stimulation of both the cochlea and the vestibular system solely by a cochlear implant (CI) seems not feasible in patients [8]. Furthermore, several studies pointed towards possible crosstalk from cochlear implants towards the vestibular nerves [9–11]. A very recent study even showed that vestibular co-stimulation, measured by E-VEMPS (vestibular-evoked myogenic potentials to electrical stimulation) with a lateral wall electrode, was present in the majority of patients [12]. Mathematical modeling plays a critical role in the development of these implants, providing a means to assess variability and performance without the need for extensive in-vivo studies [13–17]. However, existing models have predominantly focused on either cochlear or vestibular function independently. To the authors’ best knowledge, no models exist to date that consider both systems simultaneously.

Understanding the interplay between stimulation in the cochlear and vestibular organs is crucial for advancing cochlear-vestibular implant (CVI) technology. Stimulation by a CVI and predicting the interactions between cochlear and vestibular functions allows for the development of more targeted and effective treatment strategies. A novel modeling workflow has been devised in this work to simulate combined cochlear-vestibular stimulation, expanding our prior research focused on vestibular implant analysis [18–20].

As there are multiple clinical studies ongoing regarding CVIs [21–23], there is a need for mathematical modeling approaches depicting the vestibular and the cochlear system together. Modeling the inner ear nerves in combination with a realistic 3D model of the inner ear can provide clinical researchers with a tool to predict the outcome of different stimulation scenarios in human trials without testing those scenarios in vivo first. This paper aims to analyze the effect of various stimulation scenarios in combined inner ear implants. Focusing on simultaneous and interleaved stimulation scenarios, the model will be used to analyze the influence of the combined stimulation possibilities on the neural activation of the inner ear nerve fibers. The model will also be used to analyze the influence of the interstimulus gap and phase duration on the neural activation of the inner ear nerve fibers.

**Fig. 2:**
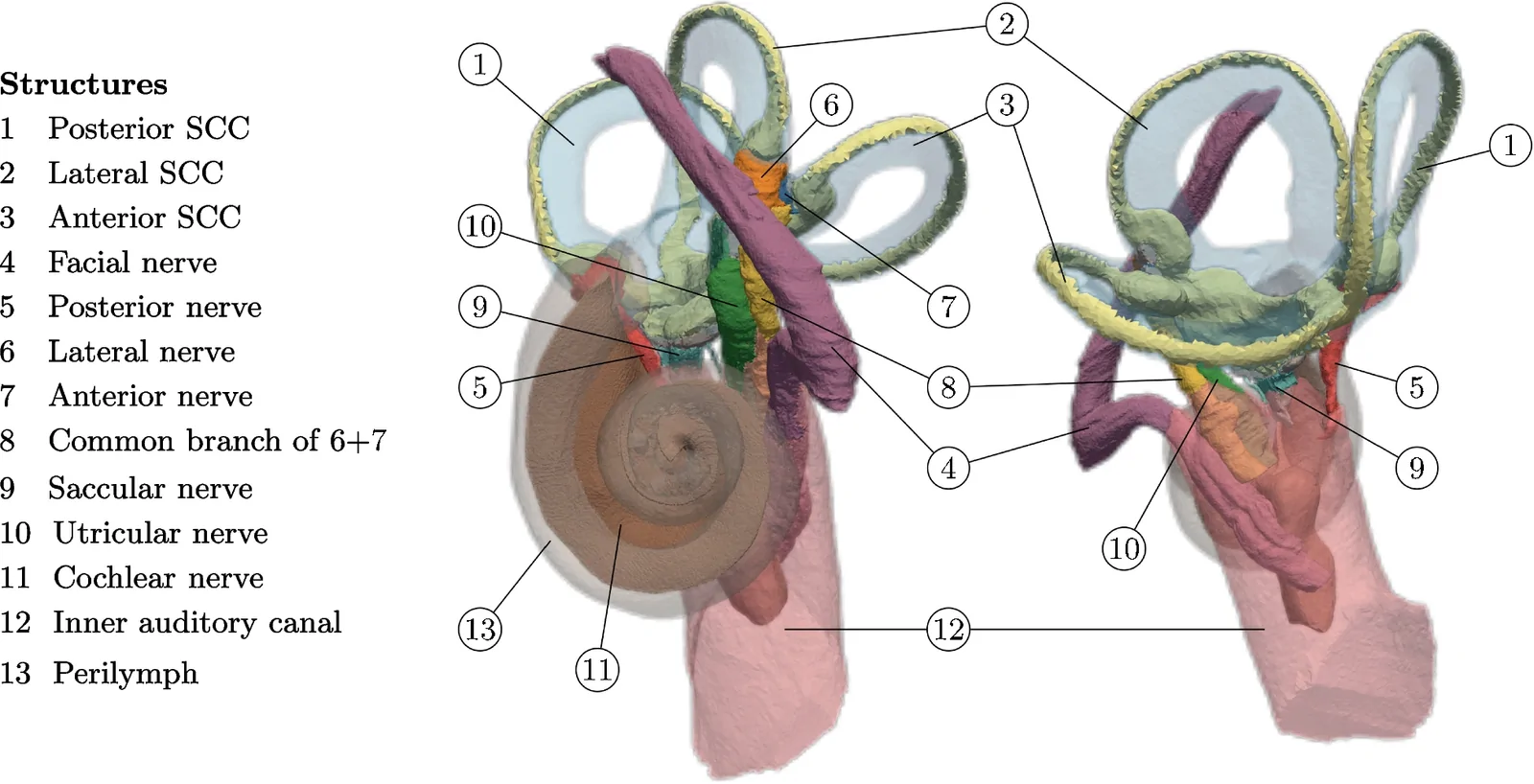
Model of the segmented inner ear with the bony and membraneous labyrinth. All relevant structures are labelled with numbers

## Materials and Methods

The established cochlear nerve model after Rattay et al. [13] was used to model cochlear nerve fibers in inner ear models previously and also used for the analysis of vestibular stimulation scenarios [18–20]. This model was then validated in our framework by comparison with the original model proposed in 2001 [13] and is planned to be further validated by patient data. The implementation and first validation were previously described in [24] and further optimized and extended with simultaneous cochlear and vestibular stimulation scenarios. The required steps of the workflow are described in the following sections.

### Modeling Workflow

Anatomical data from 50 human inner ears, scanned with contrast-enhanced high-resolution microCT, formed the basis for generating the inner ear models as described previously in [18, 19]. For the finite element method, these scans served to segment anatomical structures and were consequently meshed. The meshing was carried out using semi-automatic algorithms described by Handler et al. [18]. The model was embedded into two concentric spheres to consider the electrically highly resistive surrounding of the inner ear. Electrodes were placed inside the scala tympani of the cochlea and perilymphatic space in the semicircular canals. From these electrodes, potential distributions were calculated by solving the Poisson equation. The interpolated potential distributions are then used as input for several multi-compartment models of the inner ear nerve fibers. The workflow is depicted in Fig. 1, which is explained in more detail in the following sections.

### Finite Element Method

#### Inner Ear Model

The inner ear model is constructed utilizing preserved inner ears from human specimens donated to the Division of Clinical and Functional Anatomy of the Innsbruck Medical University by individuals who provided informed consent for scientific and educational purposes before death [25, 26]. All cadavers were preserved using an arterial injection of a formaldehyde-phenol solution / an alcohol-glycerin solution and immersion in phenolic acid in water for one to three month [27]. For detailed information on specimen preparation, readers are directed to the works of Handler et al. [18] and Johnson Chacko et al. [28].

This work employs one of several inner ear models previously generated, all derived from a voxel-based 3D images of adult human inner ears. All scans were acquired with a microCT scanner at 15 μm isotropic voxel size after contrast enhancement [29]. Segmentation and labeling encompassed the bony and membranous labyrinth, the inner ear’s nerves along with their branches, the facial nerve, and the inner auditory canal. Utilizing this 3D image data, a detailed tetrahedral mesh of a human inner ear was constructed and is depicted with all relevant structures in Fig. 2 [18, 28]. To the best of our knowledge, there exist no significant differences with respect to female/ male inner ear anatomy. The dataset used in this study was not selected based on gender but on the suitability for the development of a high-quality computer model.

Subsequently, the segmented data undergoes meshing into tetrahedrons utilizing the Computational Geometry Algorithms Library (CGAL) [30] and TetGen [31], resulting in a model with approximately 9 million tetrahedral elements. The designated components are encapsulated within a bone sphere with a diameter of 5 cm and a saline layer of 1 cm thickness to account for the neighboring structures of the inner ear, predominantly composed of bone.

#### Potential Distribution

Every tetrahedral element within the specified volume mesh is allocated an electrical conductivity property $\boldsymbol{\sigma }$ (in S/m) based on its assigned label in the segmented model and material properties from literature. Anisotropic conductivity values are accounted for nerves as previously outlined [18, 32]. The potential distribution Φ (in V) is computed for each electrode configuration with the reference potential being the outer layer of the model. Each electrode can operate as a current source, emitting anodic or cathodic currents, or as a current sink to dissipate the emitted current in a bipolar stimulation scenario. Both active and reference electrodes utilize an instrumental electrode model [20, 33]. The electrical potential distribution is then calculated by solving the Poisson equation

1 $$\begin{aligned} -\nabla \cdot (\boldsymbol{\sigma } \nabla \Phi ) = \nabla \cdot \textbf{J} = I \end{aligned}$$

with Φ (in V) being the electric potential, $\textbf{J}$ being the current density (in A/m^2^) and *I* being the current source density (in A/m^3^). Each current source is simulated to emit a unit current of 1 A across its surface. The reference potential of 0 V is established for each reference electrode within the model. Subsequently, the simulated potential distribution associated with the unitary current is scaled by the time-dependent stimulus amplitude in order to obtain the time-dependent potential distribution of the applied stimulus. This adaptation is facilitated by the assumptions of quasi-static conditions [19].

The electrical conductivities of the various tissues within the inner ear are detailed in Table 1. The peri- and endolymphatic fluids present within the cochlea, vestibule, semicircular canals, and the saline layer are presumed to exhibit high conductivity. Two conductivity values are designated for nerve tissue: one aligned with the neural pathway and another perpendicular to it, resulting in anisotropic conductivities.

**Table 1:** Electrical conductivity acquired for the finite element model, adapted from [18]. The values are based on the works of [35] ^(a)^ and [58] ^(b)^

Tissue/material	σ (S/m)
Bone	0.0139^(a)^
Nerve longitudinal	0.333^(a)^
Nerve transversal	0.0143^(a)^
Scala tympani/media/vestibuli	2.0^(a)^
Endolymph/Perilymph	2.0^(a)^
Saline layer	2.0^(b)^

### Electrode Positions

**Fig. 3:**
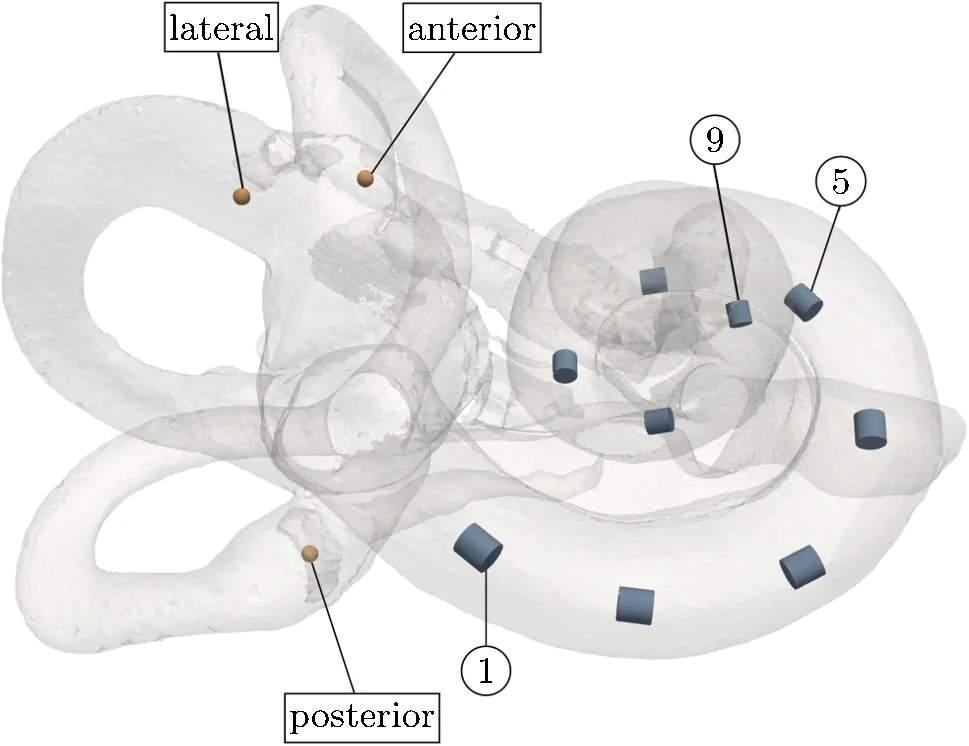
Placed electrodes inside the scala tympani of the model’s cochlea. 9 electrodes (blue) are placed along the scala tympani, with an insertion angle of roughly 720°. Each electrode is numbered from base to apex as shown. The vestibular electrodes in orange are located 750 μm from the center of the sensory epithelia of the ampullary nerves along their transversal axis

Three spherical electrodes are positioned into the perilymphatic spaces of the vestibular system within the inner ear model. These placements are determined by the central location specified by D’Alessandro et al. [20], where the electrode centers are located at a distance of 750 μm from the center of the crista ampullaris, the sensory epithelial ridge containing the hair cells. Represented as orange spheres in Fig. 3, the electrodes have diameters of 0.3 mm [17, 20].

Cochlear electrodes were considered as cylinders in the model, which were evenly distributed along a manually defined spline in the scala tympani using a distance of 2.4 mm between the centers of the cylinders. This arrangement spans approximately 720° into the cochlea, realizing a possible electrode positioning in this patient’s cochlea. The most basal electrode contact of the CI is placed 3 mm away from the round window, similar to a round window insertion commonly done in patients nowadays. The diameters of the electrodes vary from the base to the apex following one electrode geometry used by *MED-EL* [34]. At the basal end, the diameter is 0.8 mm, gradually reducing towards the helicotrema to 0.3 mm. Each electrode is assigned as an active, inactive, or reference electrode for every specified stimulation configuration. The electrodes are integrated into the model, and the meshes of the electrodes and the inner ear anatomy are merged. Electrodes comprise a finer mesh for a better consideration of the electrodes’ geometry in the meshed model [18].

### Nerve Models

#### Vestibular Nerves

Nerve fibers were created using the algorithm previously described by Schier et al. [19] and Handler et al. [18]. With this algorithm, the course of nerve fibers is generated, following the path of each ampullar nerve. Four hundred fibers are generated for each nerve branch, which are distributed according to three fiber types along the sensory epithelium. The *calyx units* represent nerve fibers innervating type I hair cells and are located mainly centrally, *bouton units* are nerve fibers that appear predominantly peripherally and innervate type II hair cells, and the *dimorphic units* are scattered over the whole sensory epithelium, innervating both hair cell types [35]. Axonal diameters of the vestibular fibers were adapted as proposed by Schier et al. [19]. The internodal distance was calculated with a linear dependency concerning the myelinated fiber diameter with a ratio of 0.7 between the axonal diameter and the diameter of the myelinated fiber [36]. The length was set to 1μm for the nodes of Ranvier. The distal heminodes were assigned a longer length of 2μm [35]. Exemplary vestibular fiber pathways are depicted in the publication of D’Alessandro et al. [20], Figure 10.

**Fig. 4:**
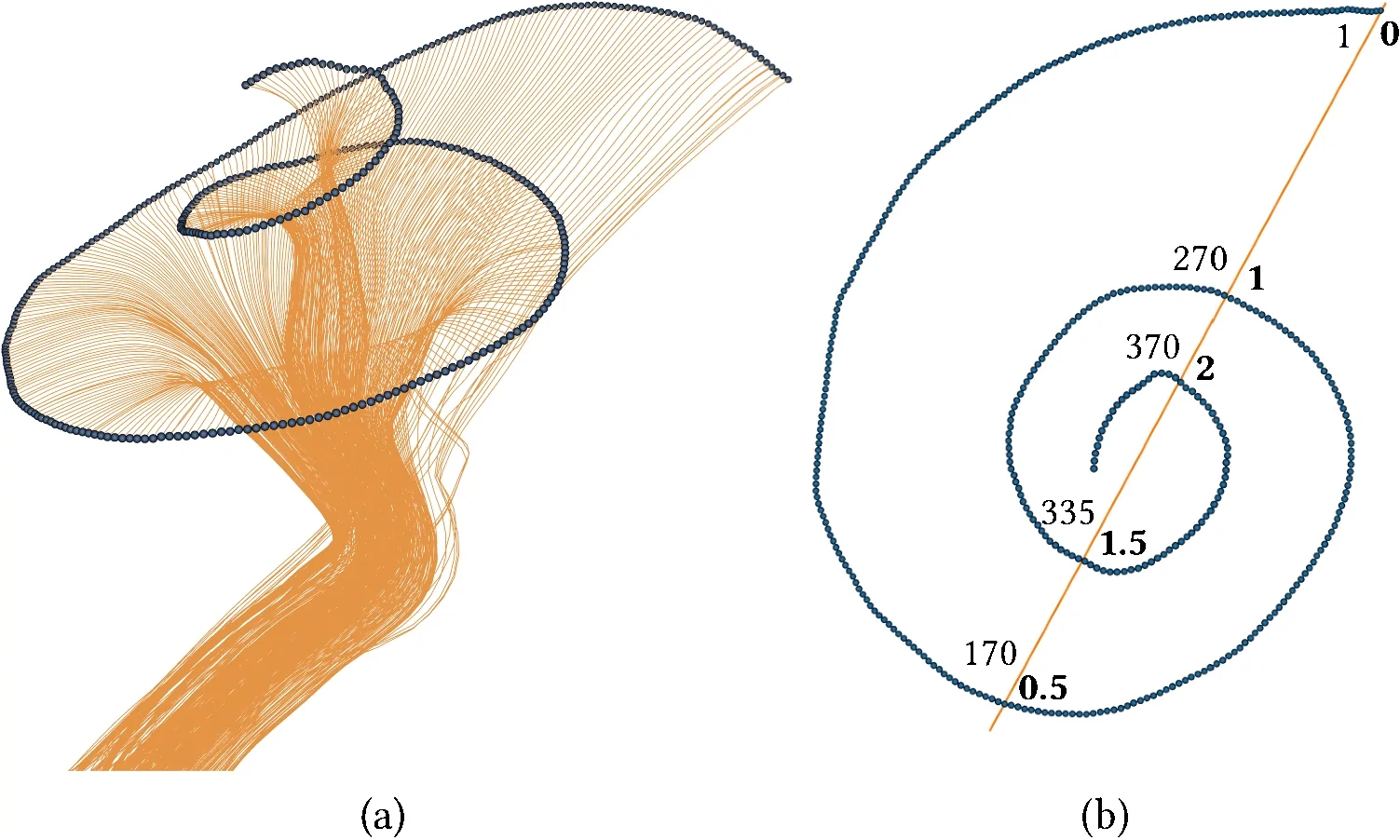
Visualization of the cochlear axons starting points at the heminodes used in this work. **a** 400 cochlear nerve fiber starting points (blue dots) with respective calculated nerve fiber paths (orange lines). The shown orange paths are not true to scale with the fiber’s diameter. **b** Horizontal plane view of the cochlea facing the central axis of the cochlear nerve. The starting points are equally spaced along the tonotopical axis. Along the reference line (orange) the respective fiber numbers are indicated together with cochlear turns (bold)

A modified SENN model proposed by Schier et al. [19] was used as an electrical neuron model for vestibular fibers. In this model, the myelin sheaths act as perfect insulators, allowing transmembrane currents to only occur at nodes of Ranvier. These currents consist of ions flowing through voltage-gated Na+ and K+ channels alongside currents occurring from membrane capacitance and leak conductance. Neighboring nodes of Ranvier along one axon are connected through axoplasmic conductances in the electrical circuit. Previously calculated potential distributions of modeled electrode configurations were interpolated at the nodes of Ranvier. The interpolated potentials were then scaled based on the stimulus strength of the studied protocols. These scaled potentials served as the extracellular input for the neuron model. The normalized coefficient of variation CV* [37] was used to consider the spontaneous firing rate of the three fiber types. For each fiber, initial values for gating variables, membrane voltage, and the values for the afterhyperpolarization were assigned from a random point in time of a generalized fiber simulation of each vestibular fiber type, using the spontaneous firing rates. This results in an offset in the percentage of activation at the zero-amplitude level [19].

**Fig. 5:**
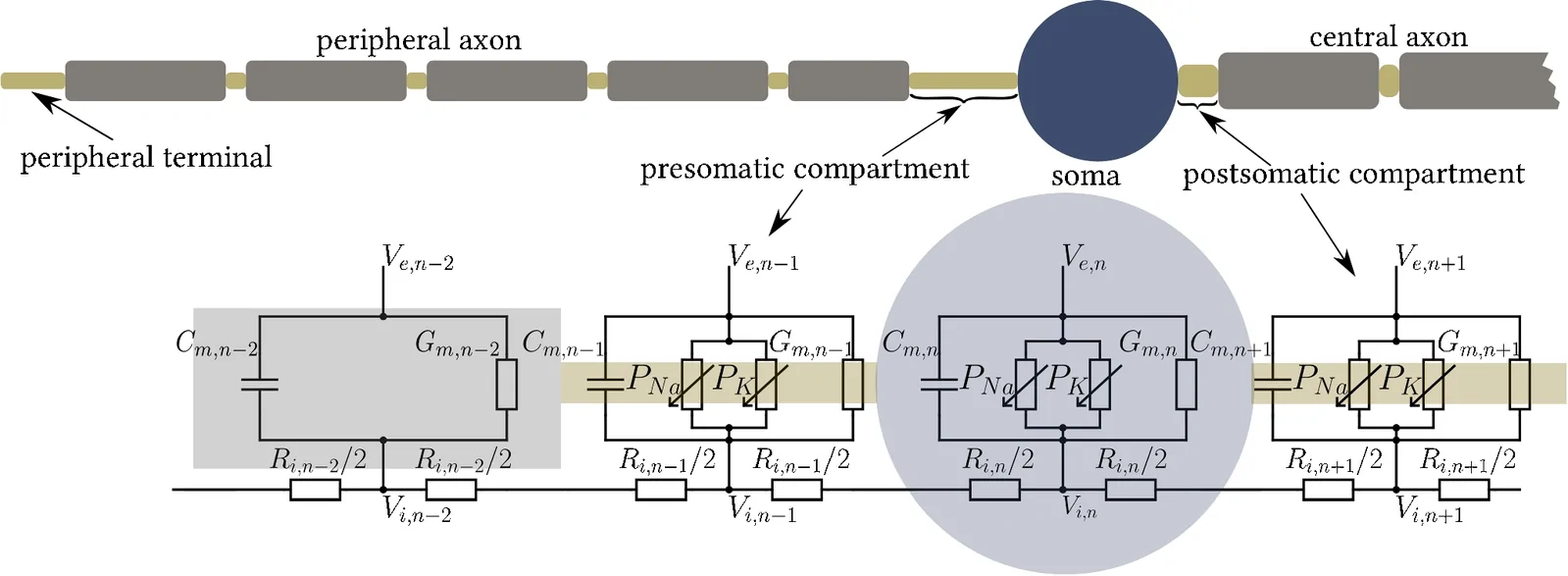
Equivalent circuit of the model after Rattay et al. [13] with active and passive compartments. Modified from [13]

#### Cochlear Nerve

A uniform distribution of the nerve fibers was considered to facilitate the location-dependent interpretation of the cochlear nerve fiber activation along the cochlear turns with the limited number of fibers in the computational model. For this, a line was placed with a semi-automatic workflow at the heminodes near the habenula perforata of the cochlear nerve. The cochlear model used in this study had a line length of roughly 33 mm with nearly 2.5 cochlear turns, which corresponds to an average cochlea of a human [38]. Based on this line, 400 equally spaced starting points in the region of cochlear heminodes where action potentials arise were generated (see Fig. 4b). Out of those points, the cochlear fibers were computed, following a possible path through the cochlear nerve (see Fig. 4a). The algorithm used is firmly based on the algorithm for the vestibular fibers of Schier et al. [17] and Handler et al. [18] and was changed for use with previously fixed starting points. This approach, with fixed starting points for the cochlear nerve fibers, enables an efficient interpretation of the excitation profiles that are later shown. It ensures a tonotopic mapping of the fibers to the cochlear turns and, therefore, to the respective frequency they encode.

All the stimulation scenarios shown in the following sections are carried out with the same set of nerve fibers, to ensure comparability across varying stimulation scenarios and interpretation questions.

The Hodgkin-Huxley model [39] is the foundational basis for the multi-compartment model developed after the work by Rattay et al. [13]. In the cochlear nerve fiber model, the underlying principle is predicated on the notion that a single nerve fiber can be delineated into multiple components, each possessing distinct geometric and electrical properties, a concept commonly referred to as neuronal cable theory [39]. These components are based on subcellular anatomical compartments such as the peripheral terminal, peripheral hemninodes/nodes and internodes, presomatic compartment, soma, postsomatic compartment, and central nodes and internodes after the transitional zone that comprise the central nervous system (CNS) part of the cochlear nerve, each characterized by a specific membrane composition tailored to its physiological function. To accurately depict the average membrane voltage of each compartment while maintaining precision, the compartment size must be sufficiently small; otherwise, for detailed representation of individual components, the compartment must be subdivided into smaller sub-compartments. The electrical circuit contains the cell membrane of each compartment and comprises capacitive, ionic, and ohmic components [13].

An exemplary equivalent circuit for a section of one nerve fiber is shown in Fig. 5. At the central point of each circuit, the flowing currents can be described with Kirchhoff’s first law as

2 $$\begin{aligned} \frac{d(V_{i, n}-V_{e, n})}{dt} \cdot C_{m, n}+ &   I_{ion, n} + \frac{V_{i, n}-V_{i, n-1}}{(R_n / 2)+(R_{n-1} / 2)}\nonumber \\+ &   \frac{V_{i, n}-V_{i, n+1}}{(R_n / 2)+(R_{n+1} / 2)} = 0 \end{aligned}$$

This equation can then be simplified using the reduced membrane voltage $V = V_i -V_e -V_{rest}$ with $V_i$ being the intracellular potential, $V_e$ the extracellular potential, and $V_{rest}$ the resting potential of the fiber. This leads to the following system of ordinary differential equations describing the time-dependent course of $V_n$ at every compartment:

3 $$\begin{aligned} \begin{aligned} \frac{dV_{n}}{dt} \!=\!\frac{1}{C_{m, n}} \cdot \left[ -I_{ion, n} + \frac{V_{n-1}-V_{n}}{(R_n / 2)+(R_{n-1} / 2)} + \frac{V_{n+1}-V_{n}}{(R_n / 2)+(R_{n+1} / 2)} \right. \\ \left. + \frac{V_{e, n-1}-V_{e, n}}{(R_n / 2)+(R_{n-1} / 2)}+\frac{V_{e, n+1}-V_{e, n}}{(R_n / 2)+(R_{n+1} / 2)} \right] \end{aligned} \end{aligned}$$

As for the first and last compartments, Eq. 3 has a reduced form as of lack of neighbors. For each compartment, the membrane surface area has to be calculated to properly consider the membrane capacitance $C_m$ of each compartment and the ion currents for each active compartment [13].

There are two parts to the ion current: A noise component that characterizes the channel current fluctuations in unmyelinated active compartments and a part consisting of voltage-specific ion channels. A computationally efficient noise technique was chosen by Rattay et al. [13] to mimic the stochastic characteristics of the auditory nerve fibers. The noise current is injected into the middle node of the active compartments as follows:

4 $$\begin{aligned} I_{noise, n} = GAUSS \cdot k_{noise} \cdot \sqrt{A_n \cdot g_{Na}} \end{aligned}$$

Here, the term *GAUSS* represents a Gaussian noise current characterized by a mean of 0 and a standard deviation of 1, with variations occurring at intervals of 2.5 μs. The parameter $k_{noise}$ serves as a universal factor across all compartments, reflecting the intensity of the noise current and directly influencing the thresholds of the fibers [40, 41]. Additionally, $A_n$ signifies the membrane area, while $g_{Na}$ represents the maximum sodium conductance specific to each compartment [13].

This methodology mitigates noise in larger fibers, as doubling the axonal diameter results in a twofold increase in ionic current while only augmenting the noise current by a factor of $\sqrt{2}$. The fiber diameter of peripheral axons across the cochlear nerves was set to 1 μm with no standard deviation. This ensures all fibers have comparable thresholds, enabling an interpretation of the location-dependent activation threshold along the cochlear nerve without the influence of randomized fiber diameters. The used values for the cochlear fibers can be found in Table 2.

**Table 2:** Parameters used for the cochlear nerve fibers in the model. Adapted from [13, 54]

Sign	Meaning	Value	Unit
$d_p$	Diameter peripheral axon	1.0	μm
$d_c$	Diameter central axon	$2 \cdot d_p$	μm
$d_s$	Diameter soma	20±2.5	μm
	Node length	2.5	μm
	Length terminal	10	μm
	Length presomatic compartment	100	μm
	Length postsomatic compartment	5	μm
$\rho _i$	Intracellular (axoplasm) resistivity	0.3	kΩ cm
$g_m$	Membrane conductivity internodes	1	mS/cm
$c_m$	Membrane capacity	1	mF/cm

**Fig. 6:**
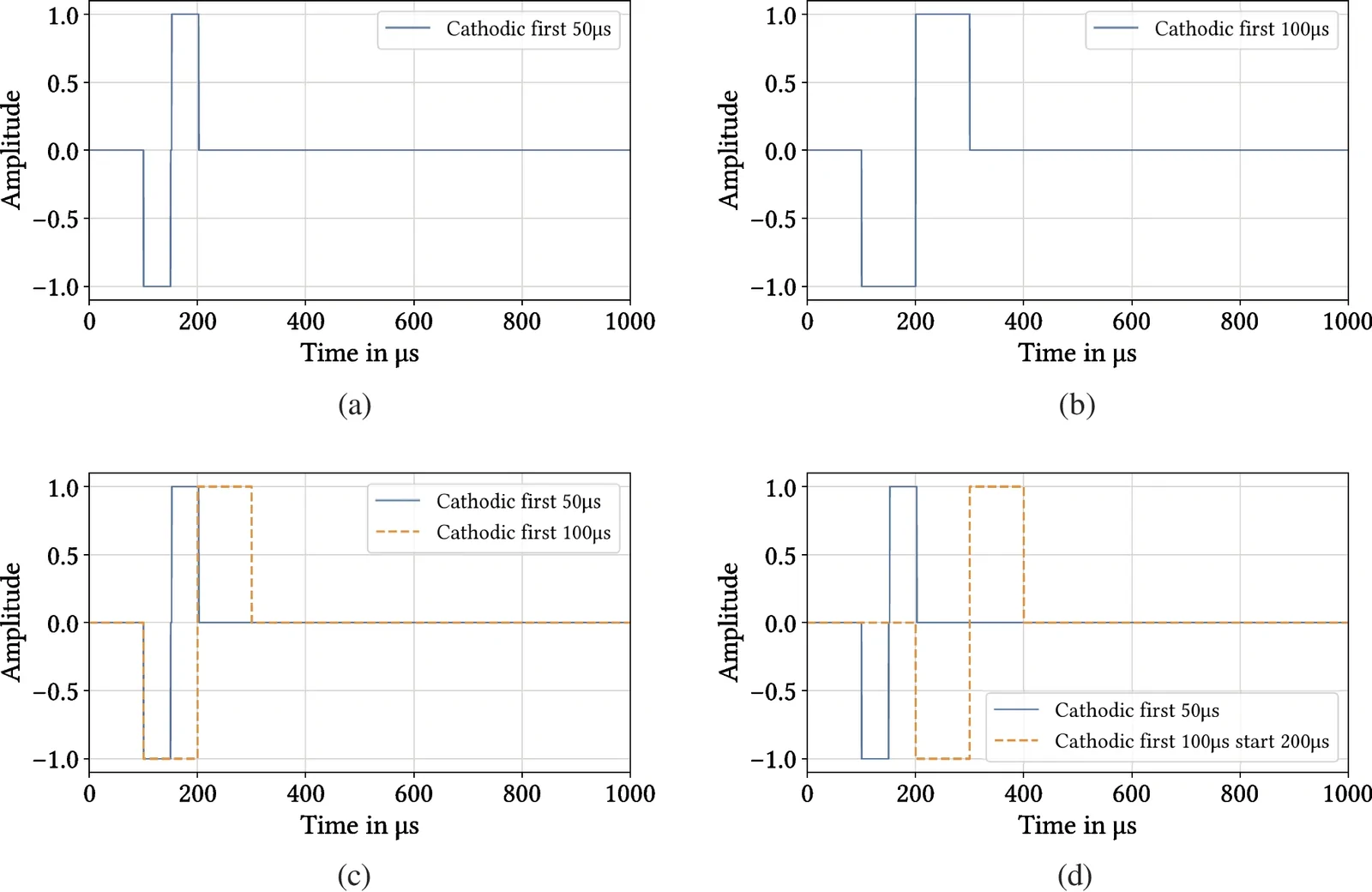
Exemplary stimulus waveforms used in the tested scenarios in this work, presented for the length of 1 ms. All used waveforms have a unitary amplitude of 1 and are scaled in the framework to apply the desired stimulus amplitude. **a** Cathodic first charge balanced pulse with a phase width of 50 μs and an IPG of 2 μs used at active cochlear electrodes. **b** Cathodic first charge balanced pulse with a phase width of 100 μs and no IPG used in active vestibular electrodes. **c** Simultaneous stimulation scenario of the cochlear and the vestibular stimulation waveform. Both waveforms start at the same time to mimic same-time stimulation in two electrodes. **d** Interleaved stimulation scenario of the cochlear stimulation waveform for one cochlear electrode in combination with a vestibular electrode. Both waveforms start with a time delay, to mimic interleaved stimulation in two electrodes. Here the time delay between the waveforms (interstimulus time) is 0 μs

As described for the vestibular fibers, initial values for gating variables and membrane voltage were assigned from a random point in time of a generalized fiber simulation for cochlear fibers. However, the applied model for the cochlear fibers does not consider varying spontaneous firing rates, and only a single fiber type was considered with the noise approach described previously.

### Stimulation Scenarios

If not otherwise indicated, a biphasic pulse with a pulse width of 50 μs and an interphase gap (IPG) of 2 μs was used in the simulations. The amplitude of the stimulation varied for the different scenarios using a binary search algorithm to find the fiber thresholds described and implemented by Schier et al. [17, 19] or manually defined amplitude ranges. These waveforms were used to scale the previously calculated potential distributions, which were interpolated at the nodes of Ranvier, and then served as input for the nerve models of each respective modeled nerve fiber as extracellular potential. Scaling was performed for each time step of the simulation.

The vestibular pulses considered in this work correspond to waveforms already used in patients, which were also previously used in the studies of Schier et al. [19] and a more recent study of Van Boxel et al. [42]. The pulses used for the vestibular stimulation were biphasic with a pulse width of 100 μs and an IPG of 0 μs (Fig. 6b). Both cathodic and anodic first pulses were considered with a focus on cathodic first pulses in this work.

The stimuli for the CI electrodes used in this work consider a more extended phase duration of 50 μs compared to phase durations of 30 μs commonly used in human CIs (Fig. 6a) [43]. This more extended phase duration accommodates for more realistic stimulation waveforms used in previous tests with CVIs and as commonly used in electrically evoked compound action potential (ECAP) measurements [22, 44]. In the “Phase Duration Influence” section, the influence of the pulse width on the nerve activation will be investigated.

Simultaneous and interleaved scenarios were considered to account for various possible stimulation scenarios in both inner ear organs. Figure 6c shows a simultaneous stimulation scenario, in which stimulation waveforms applied at a cochlear electrode and a vestibular electrode overlap in time, while Fig. 6d shows an interleaved stimulation scenario, which accounts for two stimuli carried out with a varying interstimulus time in two different electrodes in the inner ear. The interstimulus time was varied in this work between 0 μs and 100 μs to investigate the influence of the temporal distance between the stimuli on the nerve activation.

### Analyzed Stimulation Scenarios

This study investigated several realistic stimulation scenarios. Monopolar stimulation utilizes only a single active electrode per stimulation scenario. This was simulated first to establish a basis for comparison with interleaved and simultaneous stimulation scenarios that were investigated later.

The influence of simultaneous stimulation within the inner was examined, focusing on possible crosstalk between the vestibular and the cochlear organ as reported by Guinand et al. [45]. Secondly, interleaved stimulation scenarios were investigated. These target more sophisticated scenarios possible in CVI stimulation, which was previously investigated by Phillips et al. [23]. For both groups of scenarios, the electrodes of the vestibular system were combined with various electrode positions in the cochlea. The simultaneous scenarios are investigated due to the possibility of positive effects on stimulation outcomes, using electrodes in both organs simultaneously. Furthermore, the interleaved scenarios were investigated to obtain a more detailed understanding of the temporal gaps needed to ensure a high selectivity of the stimulation. Additionally, a potential increase or decrease of required stimulation thresholds by a previous stimulus in the other sensory organ was investigated.

In this work, the predicted percentage of fibers activated was used to quantify nerve activation. This value is dependent on how many of the total number of fibers of one simulated nerve branch are activated at a certain threshold current. This result is obtained by using a binary search algorithm, which identifies the required stimulus current threshold for each modeled nerve fiber.

Also, an approach initially described by Briaire and Frijns [46] utilizing the excitation profile of the cochlear nerve was applied. In this approach, the amplitude needed to stimulate a respective fiber is plotted against the position of the fiber in the cochlea. This allows for a better interpretation of active fiber ranges along the cochlea turns. It enables the analysis of different stimulation scenarios regarding their effectiveness in triggering action potentials in a specific region of the cochlea and minimizing current spread along the cochlear nerve.

## Results

### Monopolar Cochlear or Vestibular Stimulation

**Fig. 7:**
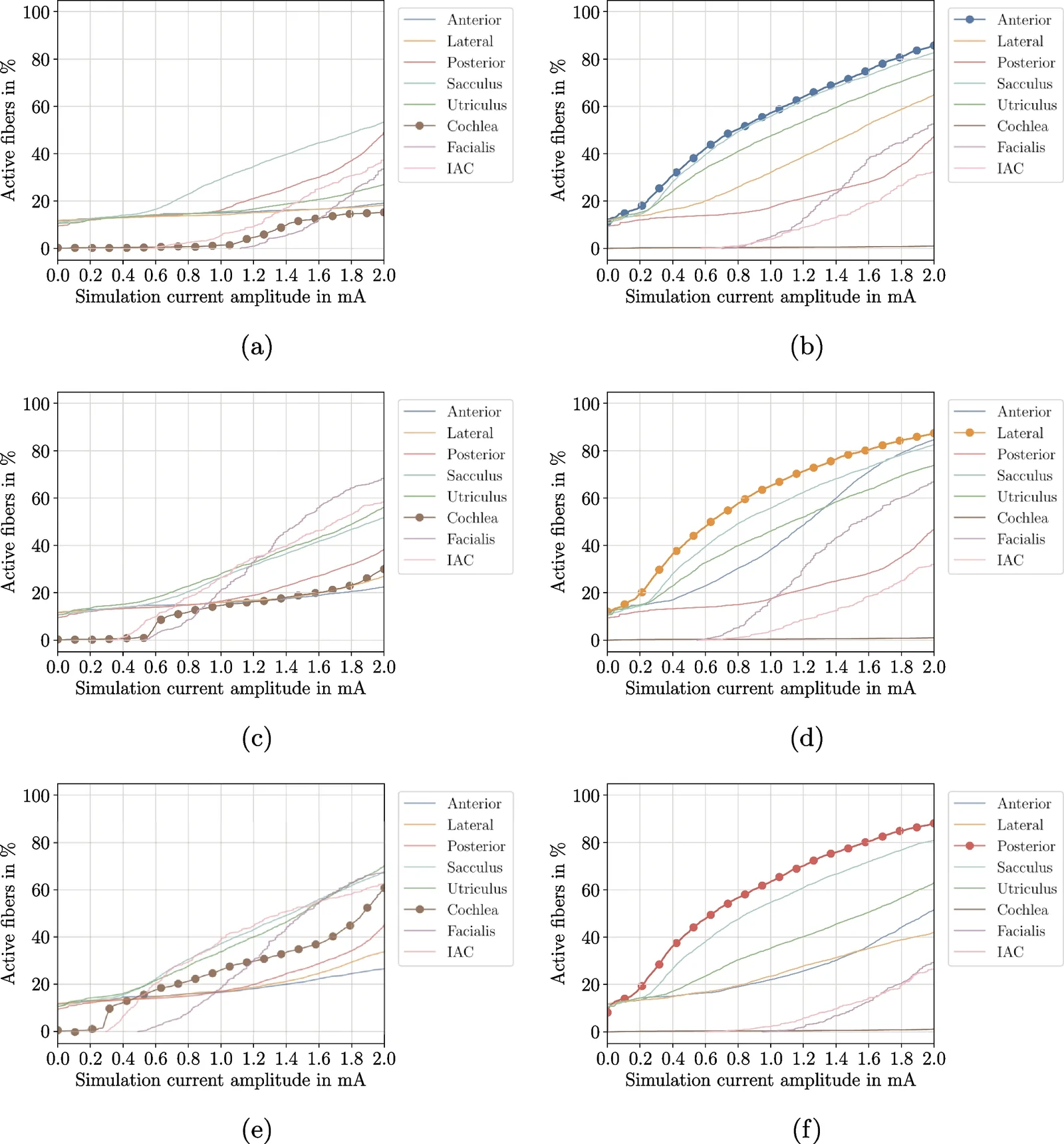
Monopolar stimulation scenarios as a basis for comparison. **a** Stimulation with the most basal cochlear electrode. **c** Stimulation with central electrode contact in the CI (position 5) located approximately at 180°. **e**: Stimulation with the most apical electrode at approximately 700° insertion. **b**, **d**, **f** Stimulation in the vestibular system, respective with the anterior (**b**), lateral (**d**) and posterior (**f**) electrode

Figure 7 shows the results of the monopolar stimulation scenarios. The monopolar stimulation scenarios are divided into two groups: one group for vestibular stimulation (right) and one group for cochlear stimulation (left). The graphs show the percentage of activated fibers in a nerve branch for a certain stimulus current applied. The activation of the targeted nerve is shown with a more saturated color, while the other nerves are shown with a less saturated color. The cochlear nerve is always depicted saturated.

For the analyzed monopolar stimulation scenarios, voltage distributions show higher peak activation levels with apical electrode placements in the cochlea compared to electrodes located in more basal regions. This results in an increase in fiber activation levels from basal (Fig. 7a) to the most apical electrode (Fig. 7e) in the cochlea.

A very high activation threshold for the cochlear nerve can be seen with vestibular electrode activation. The targeted vestibular nerve (marked with the more saturated colored lines in Fig. 7b, d, and f) shows the highest nerve fiber activation level. The saccular nerve is most cross-stimulated with all ampullar electrode stimulations, followed by the utricular nerve. Lateral canal stimulation causes more cross stimulation to the neighboring anterior canal than vice versa. This may relate to nerve fiber pathways or a bigger lumen of the lateral canal. The cochlear nerve is hardly activated by any of the ampullar electrodes alone in a reasonable amplitude range.

### Simultaneous Cochlear-Vestibular Stimulation

In the following analysis, we focus on the crosstalk during combined CVI stimulation, with only stimulation of the anterior canal electrode and selected cochlear electrode. Initially, we focused on the cochlear nerve activation when stimulating with the anterior vestibular electrode.

#### Cochlear Afferent Activation During Simultaneous CI+VI Stimulation

A stimulation threshold search considering the cochlear stimulation waveform is performed at the different cochlear electrode contacts, while simultaneously a vestibular stimulation waveform with fixed amplitude is applied via the electrode positioned in the ampulla of the anterior semicircular canal. The maximum applied current through the vestibular electrode contact was 2 mA. For each of the 400 cochlear fibers, the threshold needed for a sufficient activation is plotted against the fiber number/position in the cochlea. The fiber numbers range from 1 to 400 from the basal to the apical end of the cochlear nerve. The dashed vertical lines indicate a full cochlear turn, whereas the dotted lines signal a half cochlear turn. The results are shown in the following figures.

**Fig. 8:**
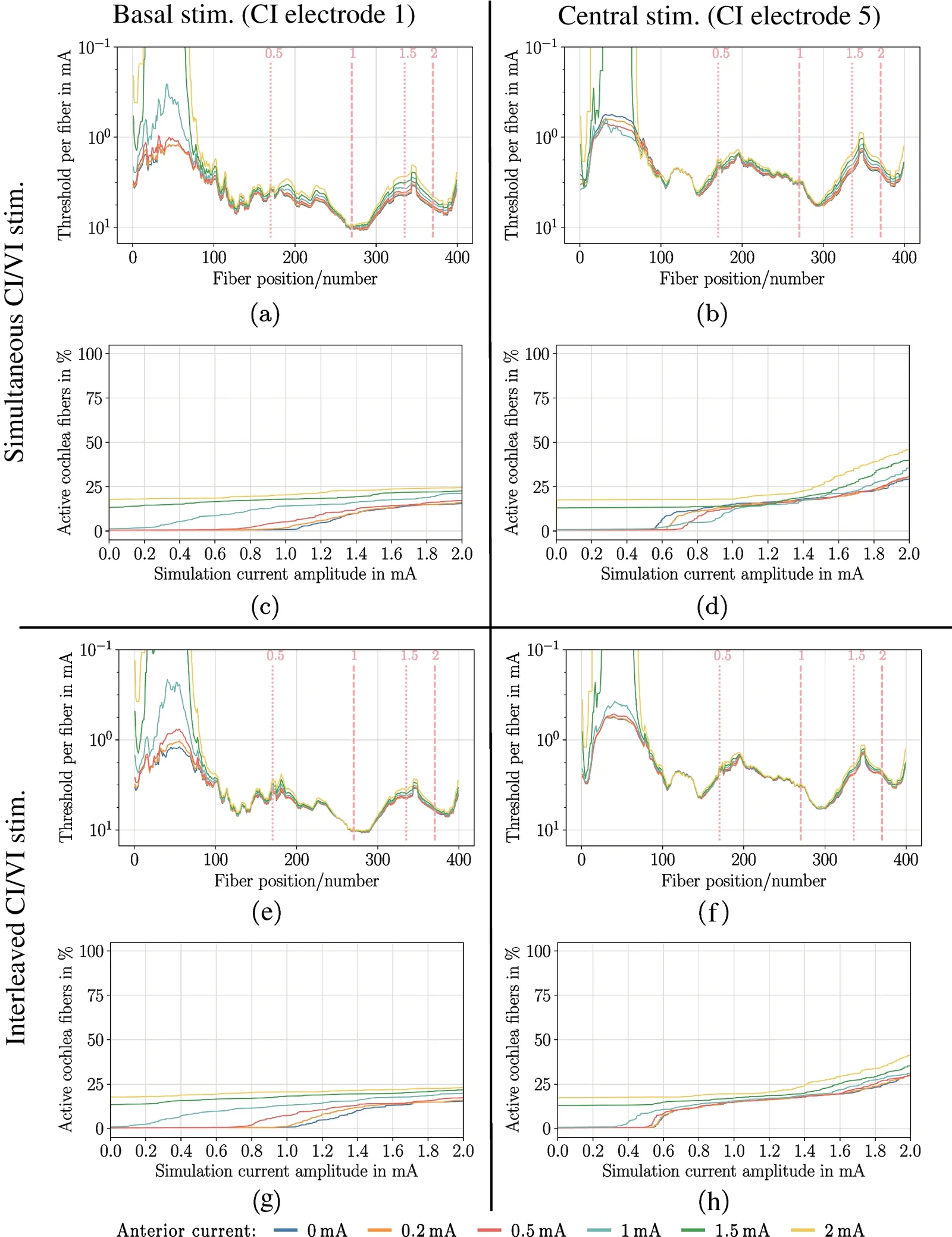
Stimulation thresholds of exemplary simultaneous and interleaved stimulation scenarios with fixed amplitude levels from 0 to 2 mA in the anterior vestibular electrode and excitation threshold search in the cochlear nerve. The left panel shows results using a basal stimulating electrode (CI electrode 1), and the right panel shows results using a central stimulating electrode contact located approximately at 180°. Displayed are cochlear activation patterns and percentages of activated fibers for simultaneous stimulation with basal/central cochlear electrodes and the anterior electrode (**a–d**) and for interleaved stimulation with basal/central cochlear electrodes and the anterior electrode (**e–h**)

A significant influence on the cochlear nerve is obvious when stimulating with the vestibular electrodes at higher amplitudes, no matter which CI electrode is active (Fig. 8a–d). The excitation profiles show lower stimulus thresholds for the fibers along the whole cochlea when stimulating with the vestibular anterior electrode at the same time. The fiber activation threshold decreases with growing vestibular stimulation amplitude. Even for stimuli up to 20% above the human applicable range (≤ 1.5 mA), a detectable influence is shown. For low stimulus amplitudes, an opposite effect can be seen. Those stimuli instead raise the current needed for activation with the more apical cochlear electrode (Fig. 8b). Growing stimulus amplitudes at the vestibular electrode do not seem to have a strong impact in the middle regions of the cochlea and show a small decrease (≤0.1 mA) in stimulation thresholds. The graphs of Fig. 8c, d show initially activated cochlear fibers from stimuli bigger than 1 mA in the vestibular electrode. Those fibers are mostly located in the basal region of the cochlea.

#### Vestibular Afferent Activation During Simultaneous CI+VI Stimulation

**Fig. 9:**
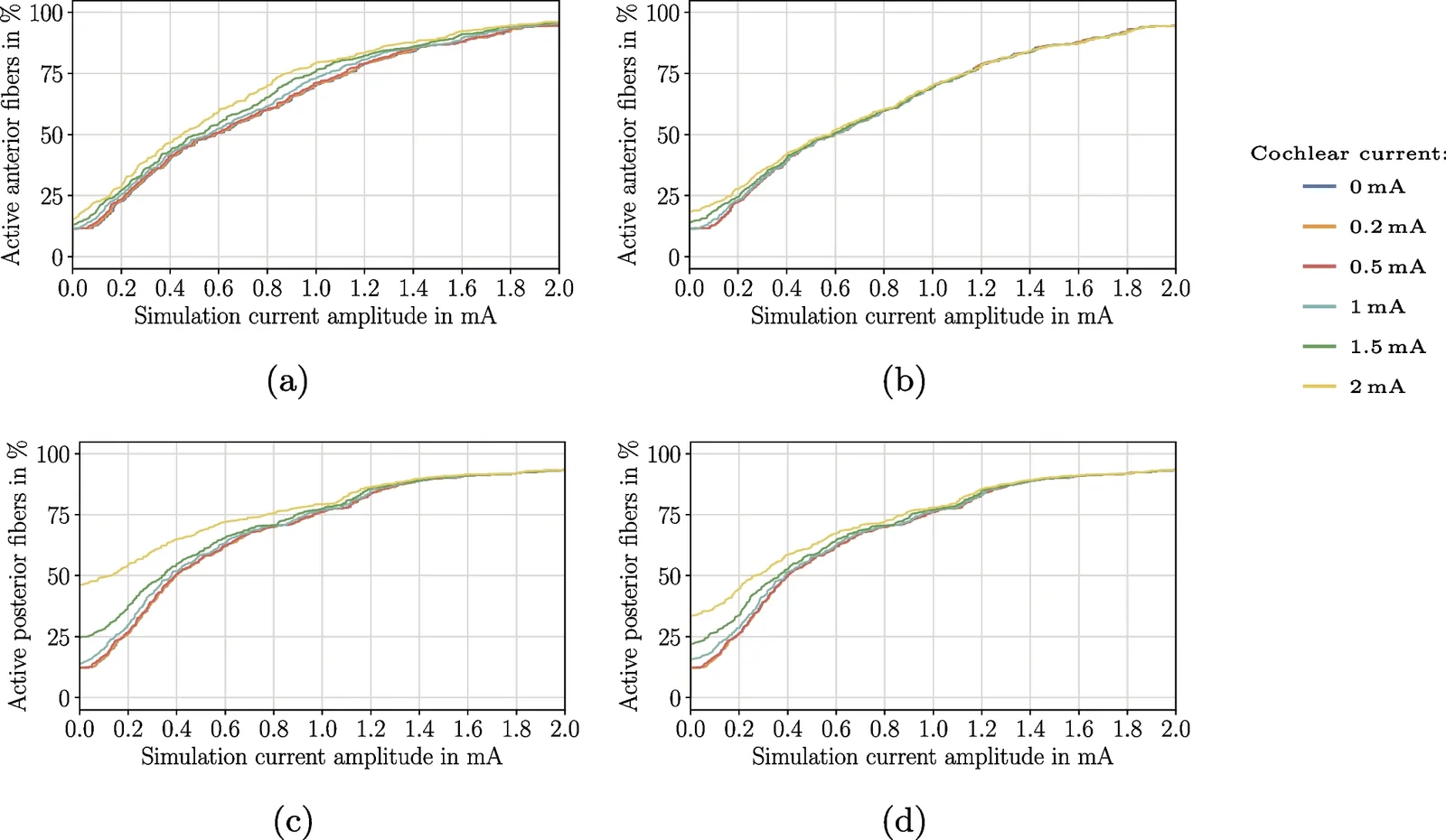
Exemplary simultaneous stimulation scenarios with two active cochlear electrodes and VI stimulation with the anterior and posterior vestibular electrode. **Top row**: Stimulation with fixed amplitude via the basal CI electrode 1 (**a**) and central CI electrode 5 (**b**) cochlear electrodes and excitation threshold search via a binary search algorithm in the anterior vestibular electrode. **Bottom row**: Stimulation with fixed amplitude in the basal CI electrode 1 (**c**) and central CI electrode 5 (**d**) cochlear electrodes and excitation threshold search via a binary search algorithm in the posterior vestibular electrode

To look at the effect of simultaneous CVI stimulation on vestibular nerve fibers, the anterior and the posterior electrodes were activated with the previously described 100 μs cathodic first biphasic pulse, respectively. The results are shown in the following Fig. 9.

Low stimulus amplitudes do not lead to notable changes in all scenarios. Therefore, the blue and orange lines are not completely visible in some graphs. With amplitudes higher than 1.5 mA, a slight influence on the activation levels of the vestibular nerves can be seen when stimulating simultaneously in both organs. This effect is more substantial when the basal cochlear electrodes are active, as can be seen due to the higher value differing from baseline of Fig. 9a and c compared to Fig. 9b and d. Crosstalk effects become stronger when the amplitude of the cochlear electrodes increases. Small increases in fiber activation levels are shown in the range of 5–10% for the vestibular nerves. This effect is bigger for the posterior vestibular nerve.

### Interleaved Vestibular-Cochlear Stimulation

#### Amplitude Dependency

Interleaved stimulation scenarios were tested for cochlear CI stimulation after a vestibular stimulus. First, the amplitude dependency of this stimulation scenario was investigated. Therefore, the stimulation amplitude of the vestibular implant (VI) was fixed for one of the electrodes, while the stimulus amplitude of the other electrode, located in the CI, was varied. The results are interpreted using the cochlear excitation pattern and nerve activation levels. The cochlear stimulus was applied directly after the vestibular stimulus in these scenarios (see Fig. 6d).

As shown in Fig. 8e–h, the rising amplitudes of the vestibular stimulus significantly influence the nerve fiber activation. The activation of cochlear nerve fibers increases with the amplitude of the stimulus in the vestibular electrodes. This is especially visible for the basal region of the cochlea, where the activation increases with the amplitude of the stimulus in the vestibular electrode activation. For a stimulation amplitude higher than 1 mA, the basal region of the cochlear nerve shows a baseline activation of the fibers before additional fibers get activated by the increasing stimulus amplitudes of the active cochlear electrode.

In contrast to the effects of simultaneous stimulation shown in Fig. 8b, where an increasing CI stimulation threshold (+0.2 mA) for basal fibers under low VI stimulation amplitudes suggests less current spread in the cochlea, it is notable that simulations showed no such effects for interleaved stimulation (see Fig. 8f). Furthermore, when considering vestibular-cochlear cross stimulation, the effects appear to be more strongly pronounced in interleaved stimulus scenarios, as overall more cochlear fibers are activated from vestibular electrodes with lower amplitudes (e.g., compare 1 mA in Fig. 8c with Fig. 8g).

#### Interstimulus Time Gap

The interstimulus time gap may be an important parameter for summation or suppression of interleaved CVI stimulation as a possible crosstalk occurs with different phase shifts. The stimulation waveforms are applied here after each other with varying time delays.

The interstimulus delay is depicted in a range from 0 to 200 μs, and all of the following results are compared to a monopolar stimulation result of the same waveform and amplitude.

**Fig. 10:**
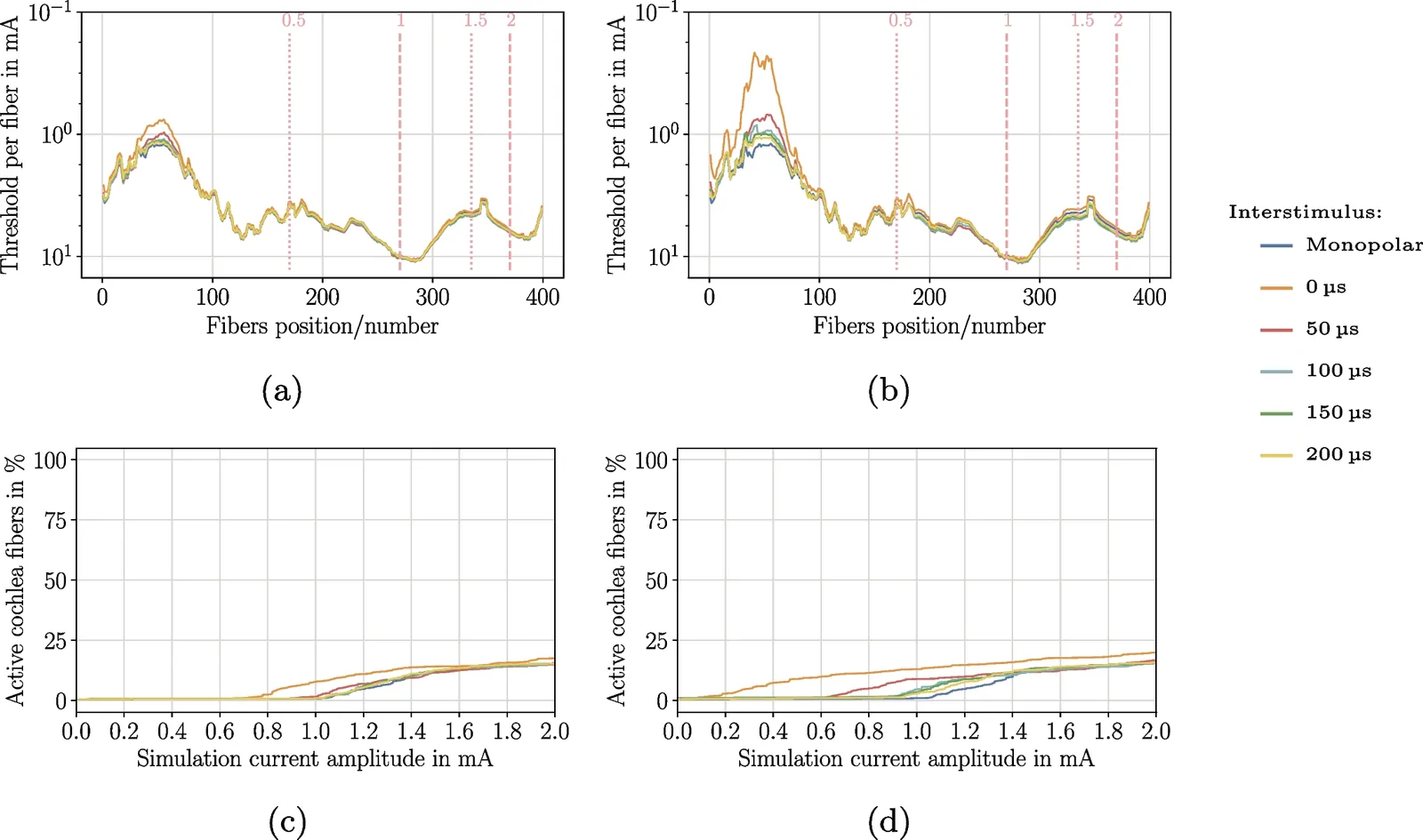
Stimulation thresholds of exemplary interleaved stimulation scenarios with varying interstimulus time gap of the CVI stimulation; the most basal cochlear electrode as well as the anterior vestibular electrodes are active. **Top row**: Stimulation with fixed amplitude in the anterior vestibular electrode and excitation threshold search via a binary search algorithm in the basal cochlear electrode. The amplitude of the anterior electrode increases from left to right from 0.5 mA (**a**) and 1 mA (**b**) stimulus amplitude. **Bottom row**: Same stimulation scenarios, depicted as percentage of fibers activated for the different interstimulus time gaps. The amplitude of the anterior electrode increases from left to right from 0.5 mA (**c**) and 1 mA (**d**) stimulus amplitude

Figure 10 shows the influence of the interstimulus time gaps of 0 to 200 in 50 μs steps on the activation levels of the cochlear nerve fibers compared to a monopolar stimulus only in the cochlea. When increasing the stimulation amplitude in the vestibular electrode contact from 0.5 mA (Fig. 10a and c) to 1 mA (Fig. 10b and d), lower stimulation thresholds and a higher percentage in active nerve fibers can be seen in the cochlea—in particular in the basal region (see Fig. 10a and b).

Furthermore, the cross-organ effect of a stimulus decreases with increasing interstimulus time, as can be seen by the higher stimulation amplitudes required for longer interstimulus gap durations, closing in on the monopolar scenarios’ threshold.

No considerable influence of CI stimulation could be shown on time-separated stimuli for the vestibular nerves. The activation levels of the vestibular nerves are not strongly influenced by the cochlear stimulus (not shown). This is true for all cochlear electrodes and all amplitudes used. These scenarios were investigated but are not further discussed in more detail, as there is no significant influence shown in the simulations.

### Phase Duration Influence

The influence of different phase durations for active vestibular electrodes on the cochlear system with simultaneous stimulation was investigated here. The phase duration was varied between 50 μs and 100 μs. The results were interpreted using the cochlear excitation pattern and nerve activation levels. These ranges were chosen, as in future CVI applications, a shortening of the vestibular stimulation length to using only intermediate phase durations could be possible. Also, the influence of using a biphasic or monophasic pulse on the vestibular electrodes was investigated. The monophasic pulse considered just the cathodic phase of the biphasic pulse defined before.

**Fig. 11:**
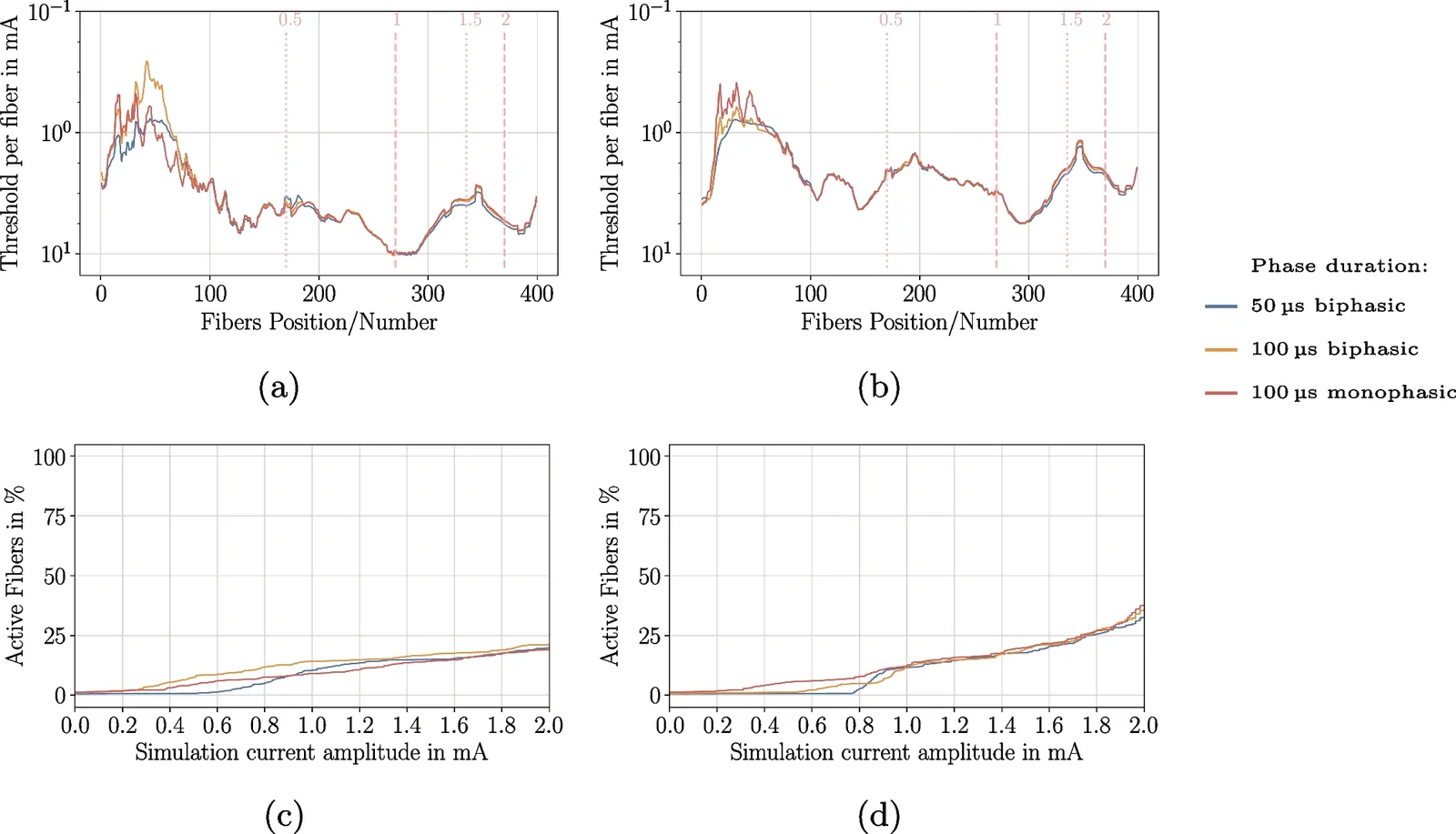
Stimulation thresholds of exemplary simultaneous stimulation scenarios with varying stimulus waveform for the vestibular anterior electrode. All scenarios here are presented for a fixed stimulus current of 1 mA for the vestibular electrode. **Top row**: Excitation profiles presented for a combination with the most basal (**a**) and the middle cochlear electrode (**b**). **Bottom row**: Excitation thresholds presented for a combination with the most basal (**c**) and the middle cochlear electrode (**d**), focusing only on the thresholds of the cochlear nerve

Significant changes are observed for shorter phases in the vestibular system, as they show a weaker influence on the cochlear nerve fibers. The amplitude of the vestibular electrode shown in Fig. 11 is fixed to 1 mA, which results in limited effects compared to higher amplitudes but serves as a sensitive scenario for phase duration changes. Nevertheless, the effects seen here become stronger with rising stimulus amplitudes above and only have a more considerable influence on cross-organ activation.

A significant influence could be shown when using the biphasic or monophasic pulse for the vestibular electrodes. In combination with the most basal cochlear electrode (Fig. 11a, c), a significant change in activation location can be seen. The biphasic pulse lowers the threshold of considerably more fibers than a monophasic pulse of the same length. This effect seems to affect only the basal part of the cochlea, as it is not as strongly pronounced or even inhibiting the cochlear fibers in the stimulation with the middle cochlear electrode (Fig. 11b, d). At more apical positions in the cochlea, just a slight change in activation was obtained when varying the stimulation waveform of the vestibular electrode contact.

## Discussion

In this work, a combined inner ear model for the human cochlea and vestibular system was established, which can be used to investigate the influence of different stimulation scenarios on the activation of nerve fibers. As pointed out earlier, these kind of models are yet missing in the landscape of literature but may significantly help in the development of CVIs.

With the model presented here, the influence of cross-organ stimulation can be successfully investigated. Furthermore, the influence of different waveform pulse widths, shapes, and interstimulus times of combined stimulation can be examined more thoroughly. Extending on a previous framework by our group, a very efficient modeling workflow is established, which can be used for further and already planned investigations.

As we used only one model, chosen for its suitability for computational modeling, we were unable to conduct a sex- and gender-based analysis in this study. To the best of our knowledge, there are no significant differences in inner ear anatomy between males and females, which should enable the results of this study to be generalizable across genders. For future studies, we plan to include more models, with an equal distribution of gender to enable the analysis of gender-based differences.

For the monopolar stimulation scenarios, the voltage distributions show higher peak activation levels with apical electrode placements, which matches several studies conducted before [47, 48]. Due to the anatomy of the used 3D model of the inner ear, there is a connection with high conductivity between the middle of the basal and the second turn of the cochlea (roughly from 70 to 200°). This high-conductivity connection in the basal region shows a strong influence during apical stimulation and influences all scenarios presented here. The rather small chosen fiber diameter can explain the relatively high cochlear threshold for the basal electrode in monopolar scenarios. While the model used in this study requires a stimulation amplitude of 1.6 mA to activate 20% of the cochlear fibers when stimulating with basal electrode contacts, other models have thresholds below 1 mA for the same percentage of activated fibers [49]. Furthermore, a change to an even more realistic electrode array model can change this seen effect due to the different geometry of the full array with isolating housing and electrode compartments. The observed non-activation of cochlear fibers in vestibular monopolar scenarios (Fig. 7b, d, f) can be explained due to the usage of a 100 μs waveform used in the interleaved and parallel stimulation scenarios of this study. In human patients, waveforms with a phase duration up to 200 μs are used [42], which doubles the charge applied during the stimulus and significantly increases the likelihood of cochlear activation during vestibular stimulation. Furthermore, our simulations indicate that mostly fibers in the basal region of the cochlea are affected by VI stimulation. During monopolar cochlear stimulation at amplitudes higher than 1 mA, the results of Fig. 7a, c, and e indicate a possible cross-activation of vestibular nerve branches. This matches recent findings that an E-VEMP is visible in most patients who have been stimulated by a lateral wall electrode [12]. Additionally, the facial nerve displays a high activation rate of up to 70% of fibers activated during cochlear stimulation (e.g., Fig. 7c), which can be explained by its anatomical proximity to the cochlea and the use of relatively large fiber diameters in the multi-compartment model. The high activation rate appears to align with human patient studies, as the facial nerve is one of the limiting factors for the upper comfortable limit in implant fitting [50]. Since a specific facial nerve fiber model has not yet been developed to the authors’ knowledge, we modeled it using the SENN model with changed fiber properties according to [19]. Integrating a detailed facial nerve model will enhance the robustness of future modeling approaches.

In the case of an implanted CVI, simultaneously activating two electrodes in the different organs is an option. Simultaneous and interleaved scenarios with varying amplitudes and time delays were investigated in the present work. The results show a significant influence of the stimulus amplitude on the nerve fibers’ activation. This effect is particularly evident in the basal region of the cochlea, where activation becomes increasingly dependent on the stimulus amplitude in the vestibular electrodes. In most scenarios, a change of 15% of activated fibers can be seen. At stimulus levels higher than 1.5 mA, the basal cochlear region even exhibits a baseline fiber activation before the cochlear electrode delivers any stimulus. Such activation patterns suggest cross-stimulation between the cochlear and vestibular systems, which should be avoided in patients. They happened exclusively in the basal part of the cochlear, indicating that auditory high frequencies could be affected with vestibular electrode stimulation. As expected, the activation of vestibular nerve fibers also increases with stimulus amplitude. For low amplitudes, no clear interaction was visible, but for amplitudes higher than 1 mA the thresholds were visibly changed, as can be seen in Fig. 9. This trend is especially pronounced in the lateral and posterior electrodes, where the activation rises strongly with increasing stimulus strength. The effects outlined here were also described previously in the work of [23], where an increased auditory effect could be seen at the ends of the cochlear array of human CVI patients. Also in the recent study of Lanthaler et al. [51], subject-specific cross-talk between the implants of the two sensory organs was found. For the basal cochlear region, one explanation may be a higher current spread, whereas the cause for the apical cochlear region is not yet clear. This also supports our findings that cross-organ stimulation is possible at higher stimulation amplitudes. For the lower current levels, there is a noticeable influence on the thresholds of the respective non-targeted nerve fibers. This effect should be tested in more detail in future work, as there is a possibility of using this in optimized stimulation protocols with multipolar stimulation scenarios.

For increased interstimulus durations in the interleaved stimulation scenarios higher than 100 μs, a decrease of activated fibers from 20 to 5% of the cochlear nerve is visible (Fig. 10). This is also true for the vestibular system, where the activation levels are not influenced as strongly by the cochlear stimulus with all cochlear electrodes in a commonly used amplitude range. Increasing the amplitude to levels that are usually above the applicable current, substantial effects on all inner ear nerves are visible.

The influence of cochlear stimuli on the vestibular nerves is smaller than vice versa. This influence was seen in both simultaneous and interleaved stimulation scenarios. One possible explanation could be different activation thresholds in the fibers of the respective organs. Multipolar stimulation scenarios with the CVI could aid the suppressing of such cross-excitation effects in the future.

For this study layout, the cochlear nerve fibers were fixated to just one diameter for all fibers to enable location-dependent comparison of nerve fiber activation thresholds without effects introduced by different nerve fiber diameters. In future studies, variable nerve fiber diameters will be taken into consideration, similar to the situation in human cochleae, as it is known that the diameter of the fibers influences the activation threshold of the nerve fibers [52]. There are some recent human data sources for getting a more realistic distribution of nerve fiber diameters [53], but to our knowledge most modeling approaches use a fixed diameter for all fibers [14, 40, 48, 54]. Innervation densities change along the tonotopic positions with the highest densities in the middle turn. Considering varying fiber densities can lead to more realistic modeling and should be incorporated into future studies [55]. The fiber diameter can also be a valuable parameter to model degenerated fiber responses [49, 56]. Simulations with a different focus were already carried out using this framework, suggesting a promising approach incorporating a more realistic fiber diameter distribution [24]. For a possible interpretation of the excitation curves with the limited number of nerve fibers, this study used fixed diameters. Limiting the number of fibers was crucial due to the direct influence on the simulation time. With new human fiber data becoming available, the model will be adapted to include a more realistic diameter distribution along the inner ear nerve fibers.

Waveform length influences the activation of the cochlear nerves, especially in the basal region. Also, the effect of a change from monophasic to biphasic pulses greatly influences the cochlea’s basal region. Considering amplitude levels higher than 1 mA, the influence of the waveform on the activation of the nerve fibers is getting more pronounced and seems noticeable for combined stimulation in the inner ear. As there were recent measurements of the effects of vestibular waveforms on nerve activation published by Van Boxel et al. [42], further waveform investigations are planned for the future, focusing on the influence of different pulse shapes and lengths on the activation of the nerve fibers. Also, the effect of commonly used pulse train stimulation will be investigated in the future, as pulse trains are a valuable tool in inner ear stimulation [23].

The position of the reference in the model has a big influence. This dependence of the stimulation on the placement of the reference electrode is well known, but up to this point, there is no clear indication in humans of where to place it to yield the best stimulation results [57]. A further investigation on this topic is planned by varying the position of the reference electrode in the model and evaluating potential effects on the nerve fiber activation.

While reasonable results based on established modeling techniques were presented in this work, validating such in silico models for the human inner ear is challenging up to that point. As there are no direct comparisons between modeled data using patients head models and measured thresholds of the same patients are possible up to this point, validation is not sufficiently right now. This model enables in-depth evaluation of scenarios that are either impossible or extremely difficult to test in humans due to ethical, practical, or technical constraints. Therefore, such a model can aid in testing new simulation scenarios and developing newly designed implants. Validation of the model with patient data is planned for the future in an ongoing project, focusing on the usage of ECAP simulation and comparison with patient telemetry data.

## Data Availability

The data that support the findings of this study are available from the corresponding author upon reasonable request.

## Code Availability

The source code for this study is available from the corresponding author upon reasonable request.

## Declarations

### Use of AI

The authors declare that no generative artificial intelligence tools were used for manuscript writing, editing, figure creation, data analysis, or any aspect of the preparation of this article. The authors declare the usage of Grammarly Proofreader for aiding the proofreading of the manuscript, which is a tool that provides grammar and spelling suggestions but does not generate content. Grammarly was used before the initial submission on August 30^th^ 2025 and before the submission of the reviewed version on May 5^th^ 2026. The authors confirm that they have reviewed the manuscript and take full responsibility for the content of this publication.

## References

[1] World Health Organization (2021) World report on hearing - executive summary. Technical report. https://cdn.who.int/media/docs/default-source/documents/health-topics/deafness-and-hearing-loss/world-report-on-hearing/wrh-executive-summary.en.pdf?sfvrsn=feb8d533_27&download=true. Accessed 18 Jan 2024

[2] Ihler F, Brzoska T, Altindal R, Dziemba O, Völzke H, Busch C-J, Ittermann T (2024) Prevalence and risk factors of self-reported hearing loss, tinnitus, and dizziness in a population-based sample from rural northeastern Germany. Sci Rep 14(1):17739. 10.1038/s41598-024-68577-3 PMC11291685 39085387

[3] Ghai S, Hakim M, Dannenbaum E, Lamontagne A (2019) Prevalence of vestibular dysfunction in children with neurological disabilities: a systematic review. Front Neurol 10:1294. 10.3389/fneur.2019.01294 PMC6928113 31920918

[4] Hackenberg B, O’Brien K, Döge J, Lackner KJ, Beutel ME, Münzel T, Wild PS, Pfeiffer N, Chalabi J, Matthias C, Bahr-Hamm K (2023) Vertigo and its burden of disease—results from a population-based cohort study. Laryngoscope Investig Oto 8(6):1624–1630. 10.1002/lio2.1169 PMC10731510 38130247

[5] Della Santina CC, Migliaccio AA, Patel AH (2007) A multichannel semicircular canal neural prosthesis using electrical stimulation to restore 3-D vestibular sensation. IEEE Trans Biomed Eng 54(6):1016–1030. 10.1109/TBME.2007.894629 PMC2767274 17554821

[6] Rubinstein JT, Bierer S, Kaneko C, Ling L, Nie K, Oxford T, Newlands S, Santos F, Risi F, Abbas PJ, Phillips JO (2012) Implantation of the semicircular canals with preservation of hearing and rotational sensitivity: a vestibular neurostimulator suitable for clinical research. Otol Neurotol 33(5):789–796. 10.1097/MAO.0b013e318254ec24 PMC3376347 22699989

[7] Santos TGT, Venosa AR, Sampaio ALL (2015) Association between hearing loss and vestibular disorders: a review of the interference of hearing in the balance. IJOHNS 04(03):173–179. 10.4236/ijohns.2015.43030

[8] De Miguel ÁR, Rodriguez Montesdeoca I, Falcón González JC, Borkoski Barreiro S, Zarowski A, Sluydts M, Falcón Benitez N, Ramos Macias A (2024) Stimulation crosstalk between cochlear and vestibular spaces during cochlear electrical stimulation. Laryngoscope 134(5):2349–2355. 10.1002/lary.31174 38010817

[9] Parkes WJ, Gnanasegaram JJ, Cushing SL, McKnight CL, Papsin BC, Gordon KA (2017) Vestibular evoked myogenic potential testing as an objective measure of vestibular stimulation with cochlear implants. Laryngoscope 127(2). 10.1002/lary.26037 27291637

[10] Wagner L, Rahne T, Plontke SK, Fröhlich L (2025) A case series shows a relation between intracochlear electric field distribution and vestibular co-stimulation with cochlear implants. Sci Rep 15(1):15976. 10.1038/s41598-025-00887-6 PMC12062236 40341611

[11] Fröhlich L, Plontke SK, Löffler LB, Manthey A, Rahne T (2024) Stimulation conditions leading to electrical vestibular co-stimulation in cochlear implant users. Laryngosc Investigat Otolaryngol 9(5):70011. 10.1002/lio2.70011 PMC11382355 39257730

[12] Fröhlich L, Mollet JK, Salha A, Eichhorn KWG, Curthoys I, Dlugaiczyk J, Wagner L, Strieth S (2026) Electrical vestibular co-stimulation by lateral wall cochlear implant electrode arrays. Otol Neurotol 47(3):493–501. 10.1097/MAO.0000000000004813 PMC12879175 41572460

[13] Rattay F, Lutter P, Felix H (2001) A model of the electrically excited human cochlear neuron: I. Contribution of neural substructures to the generation and propagation of spikes. Hear Res 153(1):43–63. 10.1016/S0378-5955(00)00256-2 11223296

[14] Alvarez F, Kipping D, Nogueira W (2023) A computational model to simulate spectral modulation and speech perception experiments of cochlear implant users. Front Neuroinform 17:934472. 10.3389/fninf.2023.934472 PMC10061543 37006637

[15] Potrusil T, Heshmat A, Sajedi S, Wenger C, Johnson Chacko L, Glueckert R, Schrott-Fischer A, Rattay F (2020) Finite element analysis and three-dimensional reconstruction of tonotopically aligned human auditory fiber pathways: a computational environment for modeling electrical stimulation by a cochlear implant based on micro-CT. Hear Res 393:108001. 10.1016/j.heares.2020.108001 32535276

[16] Kipping D, Nogueira W (2022) A computational model of a single auditory nerve fiber for electric-acoustic stimulation. JARO 23(6):835–858. 10.1007/s10162-022-00870-2 PMC9789289 36333573

[17] Schier P, Handler M, Baumgarten D, Baumgartner C (2016) Simulation and evaluation of stimulation scenarios for targeted vestibular nerve excitation. Curr Direct Biomed Eng 2(1):139–143. 10.1515/cdbme-2016-0033

[18] Handler M, Schier PP, Fritscher KD, Raudaschl P, Johnson Chacko L, Glueckert R, Saba R, Schubert R, Baumgarten D, Baumgartner C (2017) Model-based vestibular afferent stimulation: modular workflow for analyzing stimulation scenarios in patient specific and statistical vestibular anatomy. Front Neurosci 11:713. 10.3389/fnins.2017.00713 PMC5742128 29311790

[19] Schier P, Handler M, Johnson Chacko L, Schrott-Fischer A, Fritscher K, Saba R, Baumgartner C, Baumgarten D (2018) Model-based vestibular afferent stimulation: evaluating selective electrode locations and stimulation waveform shapes. Front Neurosci 12:588. 10.3389/fnins.2018.00588 PMC6125370 30214391

[20] D’Alessandro S, Handler M, Saba R, Garnham C, Baumgarten D (2022) Computer simulation of the electrical stimulation of the human vestibular system: effects of the reactive component of impedance on voltage waveform and nerve selectivity. JARO 23(6):815–833. 10.1007/s10162-022-00868-w PMC9789245 36050508

[21] Vermorken B, Volpe B, Boxel SCJV, Stultiens JJA, Hoof MV, Loos E, Soest AV, Guinand N, Fornos AP, Rompaey VV, Devocht E, De Berg RV (2022) The VertiGO! trial: study protocol for evaluating efficacy and safety of prolonged daily stimulation with a multichannel vestibulocochlear implant prototype. Preprint, In Review. 10.21203/rs.3.rs-2206722/v1

[22] Nguyen TAK, Cavuscens S, Ranieri M, Schwarz K, Guinand N, van de Berg R, van den Boogert T, Lucieer F, van Hoof M, Guyot J-P, Kingma H, Micera S, Perez Fornos A (2017) Characterization of cochlear, vestibular and cochlear-vestibular electrically evoked compound action potentials in patients with a vestibulo-cochlear implant. Front Neurosci 11:645. 10.3389/fnins.2017.00645 PMC5702472 29209162

[23] Phillips JO, Ling L, Nowack A, Rebollar B, Rubinstein JT (2020) Interactions between auditory and vestibular modalities during stimulation with a combined vestibular and cochlear prosthesis. Audiol Neurotol 25(1–2):96–108. 10.1159/000503846 PMC7050683 31968338

[24] Vey B (2024) Simulation of neural activation and electrically evoked compound action potentials during combined cochlear-vestibular stimulation. Master’s thesis, Universität Innsbruck, UMIT TIROL, Innsbruck

[25] McHanwell S, Brenner E, Chirculescu ARM, Drukker J, van Mameren H, Mazzotti G, Pais D, Paulsen F, Plaisant O, Maitre M, Laforêt E, Riederer BM, Sañudo JR, Bueno-López JL, Sprumont P, Teofilovski-Parapid G, Moxham BJ (2008) The legal and ethical framework governing body donation in Europe - a review of current practice and recommendations for good practice. Eur J Anat 12(1):1–24

[26] Riederer BM, Bolt S, Brenner E, Bueno-López JL, Circulescu ARM, Davies DC, Caro RD, Gerrits PO, McHanwell S, Pais D, Paulsen F, Sendemir E, Stabile I, Moxham BJ (2012) The legal and ethical framework governing body donation in Europe – 1st update on current practice. Eur J Anat 16:1–21. Accessed 24 July 2025

[27] Platzer W, Putz R, Poisel S (1978) Ein neues Konservierungs- und Aufbewahrungssystem für anatomisches Material. Cells Tissues Organs 102(1):60–67. 10.1159/000145619 676659

[28] Johnson Chacko L, Schmidbauer DT, Handschuh S, Reka A, Fritscher KD, Raudaschl P, Saba R, Handler M, Schier PP, Baumgarten D, Fischer N, Pechriggl EJ, Brenner E, Hoermann R, Glueckert R, Schrott-Fischer A (2018) Analysis of vestibular labyrinthine geometry and variation in the human temporal bone. Front Neurosci 12:107. 10.3389/fnins.2018.00107 PMC5834493 29535601

[29] Glueckert R, Johnson Chacko L, Schmidbauer D, Potrusil T, Pechriggl EJ, Hoermann R, Brenner E, Reka A, Schrott-Fischer A, Handschuh S (2018) Visualization of the membranous labyrinth and nerve fiber pathways in human and animal inner ears using MicroCT imaging. Front Neurosci 12:501. 10.3389/fnins.2018.00501 PMC6079228 30108474

[30] The CGAL project (2024) CGAL user and reference manual, 6.0.1 edn. CGAL Editorial Board. https://doc.cgal.org/6.0.1/Manual/packages.html

[31] Si H (2015) TetGen, a Delaunay-based quality tetrahedral mesh generator. ACM Trans Math Softw 41(2):1–36. 10.1145/2629697

[32] Handler M, D’Alessandro S, Saba R, Baumgarten D (2021) Simulation of vestibular implant stimulation in human inner ear anatomy with registered synthetic cochlear structure. In: Proceedings of annual meeting of the Austrian society for biomedical engineering 2021. Verlag der Technischen Universität Graz, Graz. 10.3217/978-3-85125-826-4-05

[33] Zhang W, Li D (2014) An instrumental electrode model for solving EIT forward problems. Physiol Meas 35(10):2001. 10.1088/0967-3334/35/10/2001 25238259

[34] MED-EL (n.d.) MED-EL cochlear implant electrode arrays. https://www.medel.pro/products/electrode-arrays Accessed 20 June 2025

[35] Hayden R, Sawyer S, Frey E, Mori S, Migliaccio AA, Della Santina CC (2011) Virtual labyrinth model of vestibular afferent excitation via implanted electrodes: validation and application to design of a multichannel vestibular prosthesis. Exp Brain Res 210(3–4):623–640. 10.1007/s00221-011-2599-x PMC3270068 21380738

[36] Hildebrand C, Hahn R (1978) Relation between myelin sheath thickness and axon size in spinal cord white matter of some vertebrate species. J Neurol Sci 38(3):421–434. 10.1016/0022-510X(78)90147-8 310448

[37] Goldberg JM, Smith CE, Fernandez C (1984) Relation between discharge regularity and responses to externally applied galvanic currents in vestibular nerve afferents of the squirrel monkey. J Neurophysiol 51(6):1236–1256. 10.1152/jn.1984.51.6.1236 6737029

[38] Biedron S, Westhofen M, Ilgner J (2009) On the number of turns in human cochleae. Otol Neurotol 30(3):414–417. 10.1097/MAO.0b013e3181977b8d 19225438

[39] Hodgkin AL, Huxley AF (1952) A quantitative description of membrane current and its application to conduction and excitation in nerve. J Physiol 117(4):500–544. 10.1113/jphysiol.1952.sp004764 PMC1392413 12991237

[40] Tanzer T (2021) Analysis of ion current fluctuations in multi-compartment models of electrically stimulated neurons. PhD thesis, Technische Universität Wien, Wien

[41] Rattay F, Tanzer T (2022) A simple model considering spiking probability during extracellular axon stimulation. PLoS ONE 17(4):0264735. 10.1371/journal.pone.0264735 PMC9022861 35446861

[42] Van Boxel SCJ, Vermorken BL, Volpe B, Guinand N, Perez-Fornos A, Devocht EMJ, Van De Berg R (2024) The vestibular implant: effects of stimulation parameters on the electrically-evoked vestibulo-ocular reflex. Front Neurol 15:1483067. 10.3389/fneur.2024.1483067 PMC11579865 39574507

[43] Strahl SB, Ramekers D, Nagelkerke MMB, Schwarz KE, Spitzer P, Klis SFL, Grolman W, Versnel H (2016) Assessing the firing properties of the electrically stimulated auditory nerve using a convolution model. In: Physiology, psychoacoustics and cognition in normal and impaired hearing. Advances in experimental medicine and biology. Springer, Cham, pp 143–153. 10.1007/978-3-319-25474-6_16 27080655

[44] Skidmore J, Ramekers D, Colesa DJ, Schvartz-Leyzac KC, Pfingst BE, He S (2022) A broadly applicable method for characterizing the slope of the electrically evoked compound action potential amplitude growth function. Ear Hear 43(1):150–164. 10.1097/AUD.0000000000001084 PMC8674380 34241983

[45] Guinand N, Van De Berg R, Cavuscens S, Stokroos RJ, Ranieri M, Pelizzone M, Kingma H, Guyot J-P, Perez-Fornos A (2015) Vestibular implants: 8 years of experience with electrical stimulation of the vestibular nerve in 11 patients with bilateral vestibular loss. ORL 77(4):227–240. 10.1159/000433554 26367113

[46] Briaire JJ, Frijns JHM (2005) Unraveling the electrically evoked compound action potential. Hear Res 205(1–2):143–156. 10.1016/j.heares.2005.03.020 15953524

[47] Saba R (2012) Cochlear implant modelling: stimulation and power consumption. PhD thesis, University of Southampton, Southampton. https://eprints.soton.ac.uk/348818/. Accessed 13 Nov 2024

[48] Nogueira W, Schurzig D, Büchner A, Penninger RT, Würfel W (2016) Validation of a cochlear implant patient-specific model of the voltage distribution in a clinical setting. Front Bioeng Biotechnol 4. 10.3389/fbioe.2016.00084 PMC5120131 27933290

[49] Croner AM, Heshmat A, Schrott-Fischer A, Glueckert R, Hemmert W, Bai S (2022) Effects of degrees of degeneration on the electrical excitation of human spiral ganglion neurons based on a high-resolution computer model. Front Neurosci 16:914876. 10.3389/fnins.2022.914876 PMC9298973 35873813

[50] Volpe B, Vermorken BL, Van Boxel SCJ, Guinand N, Pérez Fornos A, Devocht EMJ, Van De Berg R (2025) Perceptual response characterization in acute vestibular implant stimulation. J Neurol 272(11):746. 10.1007/s00415-025-13457-7 PMC12586226 41188507

[51] Lanthaler D, Griessner A, Steixner V, Corre J, Ranieri M, Cavuscens S, Grouvel G, Guinand N, Zierhofer CM, Fornos AP (2025) An investigation into possible interactions in the vestibular system and the cochlea during electrical stimulation. Front Neurosci 19:1612253. 10.3389/fnins.2025.1612253 PMC12130027 40463591

[52] Rattay F, Leao RN, Felix H (2001) A model of the electrically excited human cochlear neuron. II. Influence of the three-dimensional cochlear structure on neural excitability. Hear Res 153(1):64–79. 10.1016/S0378-5955(00)00257-4 11223297

[53] Heshmat A, Sajedi S, Johnson Chacko L, Fischer N, Schrott-Fischer A, Rattay F (2020) Dendritic degeneration of human auditory nerve fibers and its impact on the spiking pattern under regular conditions and during cochlear implant stimulation. Front Neurosci 14:599868. 10.3389/fnins.2020.599868 PMC7710996 33328872

[54] Bachmaier R, Encke J, Obando-Leitón M, Hemmert W, Bai S (2019) Comparison of multi-compartment cable models of human auditory nerve fibers. Front Neurosci 13:1173. 10.3389/fnins.2019.01173 PMC6848226 31749676

[55] Kipping D, Zhang Y, Nogueira W (2024) A computational model of the electrically or acoustically evoked compound action potential in cochlear implant users with residual hearing. IEEE Trans Biomed Eng 71(11):3192–3203. 10.1109/TBME.2024.3410686 38843064

[56] Rattay F, Tanzer T (2022) Impact of electrode position on the dynamic range of a human auditory nerve fiber. J Neural Eng 19(1):016025. 10.1088/1741-2552/ac50bf 35105835

[57] Schoo DP, Ward BK, Chow MR, Ayiotis AI, Fernández Brillet C, Boutros PJ, Lane KE, Lee CN, Morris BJ, Carey JP, Della Santina CC (2023) Vestibular implant surgery. Laryngoscope 134(4):1842–1846. 10.1002/lary.31004 PMC11458111 37767871

[58] Marianelli P, Capogrosso M, Bassi Luciani L, Panarese A, Micera S (2015) A computational framework for electrical stimulation of vestibular nerve. IEEE Trans Neural Syst Rehabil Eng 23(5):897–909. 10.1109/TNSRE.2015.2407861 25751868

